# Integrated analysis of miRNA and mRNA expression profiles in response to Cd exposure in rice seedlings

**DOI:** 10.1186/1471-2164-15-835

**Published:** 2014-10-01

**Authors:** Mingfeng Tang, Donghai Mao, Liwei Xu, Dayong Li, Shuhui Song, Caiyan Chen

**Affiliations:** Key Laboratory of Agro-ecological Processes in Subtropical Region, Institute of Subtropical Agriculture, Chinese Academy of Sciences, Changsha, 410125 China; State Key Laboratory of Plant Genomics, Institute of Genetics and Developmental Biology, Chinese Academy of Sciences, Beijing, 100101 China; Key Laboratory of Genome Sciences and Information, Beijing Institute of Genomics, Chinese Academy of Sciences, Beijing, 100029 China

**Keywords:** Cd stress, miRNA, mRNA, High-throughput deep sequencing, Rice

## Abstract

**Background:**

Independent transcriptome profile analyses of miRNAs or mRNAs under conditions of cadmium (Cd) stress have been widely reported in plants. However, a combined analysis of sRNA sequencing expression data with miRNA target expression data to infer the relative activities of miRNAs that regulate gene expression changes resulting from Cd stress has not been reported in rice. To elucidate the roles played by miRNAs in the regulation of changes in gene expression in response to Cd stress in rice (*Oryza sativa L.*), we simultaneously characterized changes in the miRNA and mRNA profiles following treatment with Cd.

**Results:**

A total of 163 miRNAs and 2,574 mRNAs were identified to be differentially expressed under Cd stress, and the changes in the gene expression profile in the shoot were distinct from those in the root. At the miRNA level, 141 known miRNAs belonging to 48 families, and 39 known miRNAs in 23 families were identified to be differentially expressed in the root and shoot, respectively. In addition, we identified eight new miRNA candidates from the root and five from the shoot that were differentially expressed in response to Cd treatment. For the mRNAs, we identified 1,044 genes in the root and 448 genes in the shoot that were up-regulated, while 572 and 645 genes were down-regulated in the root and shoot, respectively. GO and KEGG enrichment analyses showed that genes encoding secondary, metabolite synthases, signaling molecules, and ABC transporters were significantly enriched in the root, while only ribosomal protein and carotenoid biosynthesis genes were significantly enriched in the shoot. Then 10 known miRNA-mRNA interaction pairs and six new candidate ones, that showed the opposite expression patterns, were identified by aligning our two datasets against online databases and by using the UEA sRNA toolkit respectively.

**Conclusions:**

This study is the first to use high throughput DNA sequencing to simultaneously detect changes in miRNA and mRNA expression patterns in the root and shoot in response to Cd treatment. These integrated high-throughput expression data provide a valuable resource to examine global genome expression changes in response to Cd treatment and how these are regulated by miRNAs.

**Electronic supplementary material:**

The online version of this article (doi:10.1186/1471-2164-15-835) contains supplementary material, which is available to authorized users.

## Background

Cadmium (Cd) is a well-known environmental toxicant to humans and plants. Cd has been implicated as a potential cause of prostate, lung, and testicular cancer, kidney tubule damage, bone fractures, and osteomalacia that is acquired from consuming contaminated crops [1, 2]. Because of widespread Cd pollution in paddy soils and its ready accumulation in crops, people who consume Cd-contaminated foods are inevitably exposed to significant amounts of Cd [3]. Cd is a non-essential element for plants that reduces crop quality and, subsequently, food safety at low concentrations, and damages plant growth and reproduction at high concentrations. Therefore, elucidating the physiological, genetic, and molecular responses to Cd stress will be of benefit in improving both crop yield and quality.

MicroRNAs (miRNA) are a class of small non-coding RNAs (approximately 21 nt long) that bind complementary sequences in target mRNAs to specifically regulate gene expression through either mRNA degradation or translational inhibition [4]. Plant miRNAs are involved in regulating a wide range of biological processes, including signal transduction, cell identity, growth, and developmental patterning [5–7]. Furthermore, numerous miRNAs have also been reported to be involved in biotic and abiotic stress responses [8–12]. Based on microarray data, the expression of 14 stress-regulated miRNAs was observed under salt, drought, and cold stresses; miR168, miR171, and miR396 showed responses to all three stress treatments [12]. Induction of miR395 and miR399 was observed in response to sulfate and phosphate deprivation, respectively [10, 11]. Under conditions of copper (Cu) deficiency, up-regulation of miR398 expression to decrease levels of Cu/Zn superoxide dismutases 1(CSD1) and Cu/Zn superoxide dismutases 2 (CSD2) is important to ensure that the limited amount of Cu is present to support necessary biological processes. However, when exposed to high levels of Cu, the induction of *CSD1* and *CSD2* mRNA by the down-regulation of miR398 is necessary to activate antioxidant systems [8, 9]. A group of miRNAs have been identified to be Cd-responsive in rice, *Medicago truncatula*, and *Arabidopsis thaliana* [13–16]. Huang et al. [14] isolated 28 novel miRNAs from a small RNA library prepared from Cd-treated rice seedlings [14]. Ding et al. [13] identified 19 miRNAs that were induced in rice roots in response to Cd treatment in a microarray-based assay [13]. Most recently, a total of 12 Cd-responsive miRNAs predicted previously were validated using microarray assays in rice [15]. A qRT-PCR-based assay for the expression of miRNAs under Cd stress in *M. truncatula* found that miR393, miR171, miR319, and miR529 were up-regulated, whereas miR166 and miR398 were down-regulated [16].

Previous studies have investigated physiological mechanisms underlying the response to Cd stress. Cd can induce oxidative stress and activate the expression of antioxidant enzymes [17–19]. Plants produce cysteine-rich (Cys-rich) peptides that chelate Cd to form non-toxic complexes which are then sequestered into the vacuole to avoid high levels of free cytotoxic Cd in the cytosol. The enzymatically-synthesized glutathione, phytochelatins (PCs), and the gene-encoded metallothioneins (MTs) are the main Cys-rich peptides [20–22]. Using a 22 K microarray covering 21,495 genes, Ogawa et al. [23] investigated gene regulation under Cd stress in rice and found sets of genes that were induced, including cytochrome P450 family proteins, heat shock proteins, glutathione S-transferase, transcription factors, protein kinases, and some transporter genes. Herbette et al. [17] also found that genes involved in sulfur assimilation-reduction, glutathione (GSH) metabolism, and the biosynthesis of phenylpropanoids were induced during Cd stress in *A. thaliana* roots.

Independent transcriptome profiling of miRNAs or mRNAs under various stress conditions has been widely reported. In addition, several studies have reported combined analyses of sRNA sequencing expression data with miRNA target expression and/or degradome data to infer the relative activities of miRNAs associated with heavy metal stress [24, 25]. However, no such combined analysis has been reported for Cd stress in rice. Because the expression of miRNAs and mRNAs are spatio-temporally regulated independently, it remains to be elucidated how mRNA profiles change in relation to miRNA regulation in a specific tissue or organ. In addition, because the total number of miRNAs discovered in plant genomes continues to increase with advances in genomics, there are still many novel miRNAs involved in stress responses and/or developmental regulation to be identified.

To acquire a deep understanding of the changes in the transcriptome that occur in response to Cd stress in rice, we used high-throughput sequencing technology to simultaneously analyze miRNA and mRNA expression profiles in Cd-stressed rice seedlings. We combined these two datasets through two online databases and identified a total of 16 miRNA-mRNA interaction pairs in root, including six new miRNA candidates and their targets, exhibiting inverted patterns of relative expression. These high-throughput expression data provide a valuable resource to examine global genome expression changes in response to Cd treatment and how these are regulated by miRNAs.

## Methods

### Plant growth conditions and treatments

Seeds of the rice cultivar Nipponbare (*Oryza sativa* L. ssp *japonica* cv. ‘Nipponbare’) were surface sterilized with 3% sodium hypochlorite, rinsed five times with distilled water, immersed in distilled water for two days, and then allowed to germinate for another two days at 37°C. Seedlings were grown in half-strength rice growth nutrient solution under a 13-h light (28°C)/11-h dark (25°C) photoperiod. Seven-day-old seedlings were exposed to treatments with and without 60 μM CdCl_2_, and the roots and shoots were then collected separately after 6 h [13, 15, 17]. The collected samples were frozen in liquid nitrogen immediately and stored at -80°C until use.

### RNA isolation

Total RNA was isolated using TRIzol® reagent (Invitrogen, Carlsbad, CA, USA). The RNA quality was assessed on agarose gels and the concentration was determined with a NanoDrop spectrophotometer (ND-1000, NamedropsTechnologies, Wilmington, DE, USA).

### MiRNA sequencing and analysis

Four small RNA libraries were constructed, amplified, and sequenced as previously described [26–28]. The samples treated with CdCl_2_ were called CR and CS, where ‘R’ indicates the root tissue, and ‘S’ indicates the shoot tissue. The control samples, which were not treated with CdCl_2_, were called KR and KS, respectively. To evaluate the reproducibility of the data, we constructed another library from root tissue treated with CdCl_2_ and called it CR2. Thus, we had a total of five libraries in our analysis: KR, CR, KS, CS, and CR2. Small RNAs of 18–30 nt was gel-purified, 5’ and 3’ adaptors were ligated sequentially to the small RNAs, and reverse transcription was then performed. The amplified fragments were sequenced on an Illumina Hiseq™ 2000 instrument at BGI Tech in Shenzhen, China, according to the manufacturer’s protocol.

After removing the adaptor sequences, low-quality tags, contaminants, and reads shorter than 18 nt, the clean reads in the five libraries were mapped to the rice genome using SOAP2[29]. rRNA, scRNA, snoRNA, snRNA, tRNA, exon, intron, and repeats sequence tags were removed based on Rfam(10.1) database(http://rfam.sanger.ac.uk/) [30] and NCBI Genbank database (http://www.ncbi.nlm.nih.gov/) searches. Conserved miRNAs were identified through a Blastn search against the miRNA database, miRBase 19.0 (http://www.mirbase.org/) [31, 32]. For new miRNA candidates, we used the miRNA prediction software Mireap (http://sourceforge.net/projects/mireap/) [33]. For predicting the targets of new miRNA candidates, we used a plant target prediction tool available in the University of East Anglia (UEA) sRNA toolkit (http://srna-workbench.cmp.uea.ac.uk/) [34].

### Differential gene expression (DGE) library construction and Illumina sequencing

The DGE libraries for five samples were processed in parallel using Illumina sample preparation kits. Briefly, mRNA was captured from total RNA of each sample with magnetic oligo (dT) beads. Following first and second strand cDNA synthesis, Endonuclease *Nla*III was used to digest the bead-bound cDNA, and bound fragments containing a CATG sequence site adjacent to the poly (A) tail at the 3’ end were acquired. After precipitation of the 3’ cDNA fragment, Illumina adaptor 1 was added to the 5’ end; this adaptor contains a recognition site for the endonuclease *Mme*I to cut 17 bp downstream of the recognition site (CTAG) and produce 17 bp tags with adaptor 1. Illumina adapter 2 was introduced at the site of *Mme*I cleavage after removing the 3’ fragment via magnetic bead precipitation. The tags with both adapter 1 and adapter 2 were then prepared for Illumina DNA sequencing [35].

### Identification of differentially-expressed genes

Before comparing the differential expression of genes in response to Cd treatment, normalized gene expression levels were obtained by normalizing the number of raw clean tags in each library to the number of transcripts per million clean tags (TPM). A rigorous algorithm method was performed for the differential expression detection of genes across samples. A combination of FDR < 0.001 and the absolute value of log_2_Ratio ≥ 1 were used as the threshold to determine the significance of differentially-expressed genes. GO and pathway enrichment analysis were based on the agriGO (http://bioinfo.cau.edu.cn/agriGO/index.php) [36] and KEGG pathways (http://www.genome.jp/kegg/) [37, 38]. Cluster analysis was performed with CLUSTER3.0 and viewed with the TREEVIEW software program (http://rana.lbl.gov/EisenSoftware.htm) [39].

### Quantitative reverse transcription polymerase chain reaction (qRT-PCR) analysis of gene expression

To validate the sequencing data, we first randomly choose 20 differentially-expressed mRNAs and 10 miRNAs for quantitative real-time RT-PCR (qRT-PCR) analysis (Additional file 1: Figure S2; Additional file 2: Table S10). We then validated the expression data of 41 key Cd-responsive genes including transcription factors, kinase, and metabolic enzymes by the same method (Additional file 2: Table S10). For mRNA quantification, after acquiring high quality total RNA, SuperSript™II Reverse Transcriptase (Invitrogen, USA) and Oligo(dT) primers were used to synthesis first-strand cDNA. The qRT-PCR were performed using gene-specific primers (Additional file 3: Table S11) in a total volume of 20 μL as follows: 10 μL SYBR Premix Ex Taq™ Perfect Time(TaKaRa, Japan), 0.4 μL ROX Reference Dye, 4 μL primer mix (1:1 mix of forward and reverse primers at 2.5 μmol/μL each), 5.6 μL of a one-third dilution of the cDNAs as template. The reaction conditions were: 30s at 95°C followed by 40 cycles of 30s at 95°C, and 30s at 60°C. The rice UBC was used as an internal standard. For mature miRNA quantification, the miScript II RT kit was used to reverse transcribe mature miRNAs according to the manufacturer’s instructions (Qiagen, Germany). The miScript SYBR Green PCR kit (Qiagen), containing QuantiTect SYBR Green PCR Master Mix and the miScript Universal Primer with the miRNA-specific forward primer (Additional file 3: Table S11) was used to quantify mature miRNAs. The rice U6 RNA was used as the internal control for RNA template normalization. All mRNA and miRNA relative expression levels were calculated by the comparative Ct method. At least three independent biological replicates were used for each gene.

## Results

### Construction and sequencing of small RNA libraries

**Figure 1:**
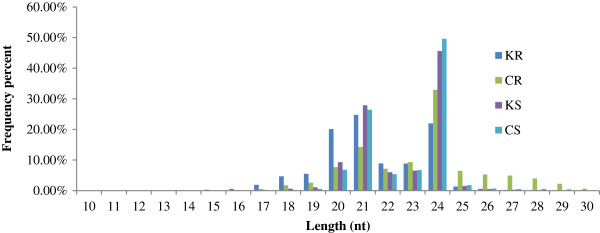
**Length distribution of tags in the small RNA libraries.**

A total of four small RNA libraries were constructed with root and shoot tissues from 7-day-old rice seedlings exposed to solutions with and without 60 μM CdCl_2_ for 6 h. We obtained 21,390,618, 21,765,186, 17,596,690, and 21,791,505 high quality reads from the KR, CR, KS, and CS libraries, respectively (Additional file 4: Table S1). After removing the adaptor sequences, low-quality tags, contaminants, and reads of <18 nt, a total of 20,686,970, 21,514,502, 17,453,217, and 21,688,526 clean reads remained from the KR, CR, KS, and CS libraries, respectively (Additional file 4: Table S1). We then aligned all reads against the rice genome using SOAP2; 14,047,474, 11,365,132,16,402,547, and 20,305,183 reads from the KR, CR, KS, and CS libraries, respectively, gave perfect matches to the rice genome sequence, representing 67.9%,52.83%, 93.98%,and 93.62% of the total reads in the four libraries. The distribution of small RNAs among the different categories is summarized in Additional file 5: Table S2. The un-annotated reads comprised most of the total reads and accounted for 30 ~ 50% of the total reads in the four libraries. Approximately 0.13% (KR), 0.1% (CR), 0.14% (KS) and 0.12% (CS) of the unique reads matched miRNAs. The length distribution of the small RNAs ranged from 10 to 30 nt (Figure 1). In the four libraries, 24 nt and 21 nt small RNAs were the main size classes and accounted for about 50% of the population, followed by 22 nt and 23 nt small RNAs.

### MicroRNA profiling of rice under Cd stress

**Figure 2:**
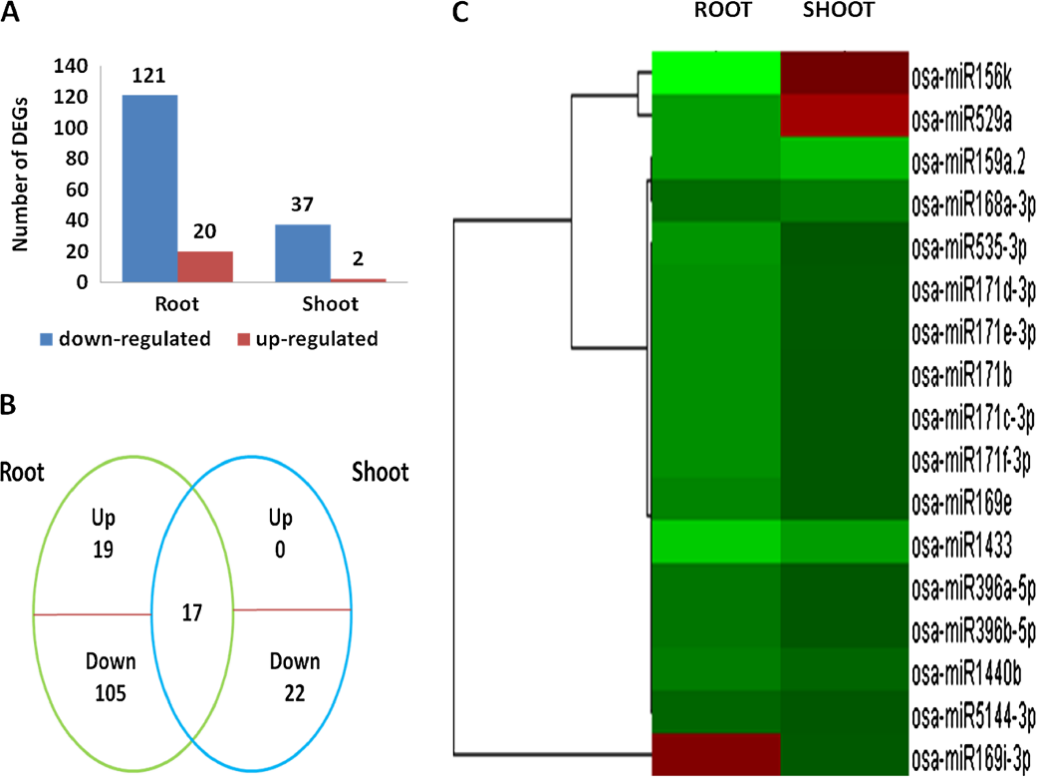
**Differentially-expressed miRNA genes in the root and shoot. (A)** The number of genes up- or down-regulated by Cd treatment by >2-fold in root and shoot (P < 0.05); **(B)** A Venn diagrams showing the unique and shared regulated miRNA genes in the rice root and shoot under Cd stress; **(C)** Hierarchical cluster analysis of 17 miRNA genes that are regulated in both the root and shoot. The relative fold-changes were analyzed. The fold-change ratios of the genes are indicated by the different colors.

The expression of miRNAs in the Cd-treated and control groups was shown by calculating the log_2_Ratio. Using |log_2_Ratio| ≥1 and *P* <0.05 as the cut-off, we identified 163 differentially expressed known rice miRNAs in our pair wise comparisons, including 121 down-regulated and 20 up-regulated in root (CR/KR), and 37 down-regulated and two up-regulated in the shoot (CS/KS) (Figure 2A and Additional file 6: Table S3 and Additional file 7: Table S4). A Venn diagram (Figure 2B) showed that the relative expression of 17 miRNAs changed in both the root and the shoot; all 17 were down-regulated except for miR156k and miR529a in the shoot and miRNA169i-3p in the root, which were up-regulated (Figure 2C).

In addition to analyzing the expression of known miRNAs, we predicted new miRNA candidates using the miRNA prediction software Mireap (http://sourceforge.net/projects/mireap/) [33]. By exploring the secondary structure, Dicer cleavage sites, and the minimum free energy of the un-annotated small RNA tags that could be mapped to rice genome, we predicted 137, 69, 154, and 165 new miRNA candidates from the KR, CR, KS, and CS libraries, respectively (Additional file 8: Tables S5). Using the same criteria (|log_2_Ratio| ≥1, P <0.05) to determine differentially-expressed miRNAs, we found eight differentially-expressed miRNAs in the root and five differentially-expressed miRNAs in the shoot (Table 1). The precursor sequences of these new miRNA candidates varied from 77 to 260 nt in length, and they formed proper predicted secondary hairpin structures with MEFIs ranging from -27.6 to -149 kcal/mol (Additional file 9: Table S6). Except for three of the new miRNA genes that are located within genes, all the others are situated in intergenic regions (Additional file 9: Table S6). Most of the mature miRNA sequences are in the 5’ arm of the stem-loop sequences, and only three miRNAs were found in the 3’ arms. As for expression patterns, one of the eight differentially-expressed root miRNAs and three of the five differentially-expressed shoot miRNAs were up-regulated, while all of the others were down-regulated.

**Table 1:** **New miRNA candidates differentially expressed in root and shoot under Cd stress**

Name	Chromosome	Arm	Mature sequence(5′-3′)	L(nt)	log2 Ratio	P-value
miRR1	6	5p	AGAAGAGTGGGAACGTGGGCTT	22	-1.59	0.00
miRR2	3	5p	AGGCGGCGGGGTGGGTGACGGT	22	-1.39	0.01
miRR3	2	5p	AGGCGGGGAGACCGGCGAGCA	21	-1.13	0.01
miRR4	7	5p	ATTGAGGAGATTGGGAAGATT	21	-1.3	0.00
miRR5	3	3p	CATGTTTGGGGATGGAGGTAG	21	-1.69	0.00
miRR6	11	3p	CTTTGAGTAGGGTCTAAACAGAG	23	2.06	0.00
miRR7	12	5p	GTGGGGCGGCGGTGGTGGCGG	21	-1.08	0.00
miRR8	2	5p	TAAAGGAAGAAGAGAGAGAGT	21	-2.12	0.00
miRS1	1	5p	CGGCGTCGTCTAGGCCGAGCGG	22	1.49	0.02
miRS2	11	3p	CGTGGTGCGGTGCGGCGGCGG	21	1.21	0.04
miRS3	6	5p	GACGGAGGGAGTAGAGTAGAAGA	23	1.4	0.03
miRS4	6	5p	TCGCCGCGGCTGGCATCAGCA	21	-1.17	0.01
miRS5	6	5p	TGCAGCTGACATGGCATGCCA	21	-1.51	0.00

### Global mRNA expression profiles in the rice root and shoot in response to Cd stress

In order to identify all miRNA targets that are differentially expressed in response to Cd stress, we used the Solexa Genome Analyzer to perform high-throughput Tag-seq analysis on rice root and shoot RNA libraries. These libraries were constructed from the same 7-day-old rice seedlings that were exposed to 60 μM CdCl_2_ for 6 h (and the control solution without CdCl_2_) and the same total RNAs were used for small RNA sequencing. The major characteristics of these four libraries are summarized in Table 2. Approximately 5.7 to 6.1 million total sequence tags per library were obtained, with 20,000 to 70,000 distinct tag sequences. Approximately 5.3 to 5.9 million total clean sequence tags per library, with 1.1 to 2.3 million distinct clean tag sequences, were produced after filtering out low-quality tags, unexpected-length tags, and single-copy tags. Finally, we obtained 187,698, 218,366, 110,103 and 114,495 unique tags for the KR, CR, KS, and CS libraries, respectively. Saturation analysis was applied to estimate whether or not new unique tags can be detected with increases in the total number of tags. As shown in Additional file 10: Figure S1, the number of unique tags increased with the total number of tags and reached a plateau shortly after 1 million tags; no new unique tag was identified as the total number approached 2 million. Therefore, the four libraries are full representations of transcripts under the different treatments.

**Table 2:** **Categorization and abundance of tags**

		KR	CR	KS	CS
**Raw data**	**Total**	5786951	5890800	6035079	5775616
	**Distinct tags**	518942	628484	252376	258567
**Clean tags**	**Total number**	5454707	5479746	5890736	5629573
	**Distinct tag numbers**	187698	218366	110103	114495
**All tag mapping to gene**	**Total number**	3814923	3717042	4938104	4600158
	**Total% of clean tags**	69.94%	67.83%	83.83%	81.71%
	**Distinct tag numbers**	75809	80554	70594	72224
	**Distinct Tag% of clean tags**	40.39%	36.89%	64.12%	63.08%
**Unambiguous tag mapping to gene**	**Total number**	2401629	2399312	2650513	2448845
	**Total% of clean tags**	44.03%	43.79%	44.99%	43.50%
	**Distinct tag numbers**	49474	53274	44370	45456
	**Distinct Tag% of clean tags**	26.36%	24.40%	40.30%	39.70%
**All tag-mapped genes**	**Number**	32683	33502	31136	31129
	**% of ref genes**	49.27%	50.50%	46.94%	46.92%
**Unambiguous tag-mapped genes**	**Number**	16348	16888	14858	14830
	**% of ref genes**	24.64%	25.46%	22.40%	22.36%
**Mapping to genome**	**Total number**	574449	555079	491620	458005
	**Total% of clean tags**	10.53%	10.13%	8.35%	8.14%
**Unknown tags**	**Total number**	1065335	1207625	461012	571410
	**Total% of clean tags**	19.53%	22.04%	7.83%	10.15%

We annotated the sequence tags based on Os-Nipponbare-Reference-IRGSP-1.0 (http://rice.plantbiology.msu.edu/index.shtml) [40], and only the clean and unambiguous tags that matched perfectly or had only a single mismatch was analyzed further. Based on these criteria, 49,474 (26.36% of the clean tags), 53,274 (24.40% of the clean tags), 44,370 (40.30% of the clean tags) and 45,456 (39.70% of the clean tags) of the tags in the KR, CR, KS, and CS libraries, respectively, were mapped unambiguously to the reference genes. Also, 555,079 (10.13% of the clean tags), 574,758 (10.69% of the clean tags), 491,620 (8.35% of the clean tags) and 458,005 (8.14% of the clean tags) unambiguous tags from these same libraries were matched to the reference genome database. However, 1,065,335 (19.53% of the clean tags), 1,207,625 (22.04% of the clean tags), 461,012 (7.83% of the clean tags) and 571,410 (10.15% of the clean tags) did not map to the reference database from the KR, CR, KS, and CS libraries, respectively (Table 2).

**Figure 3:**
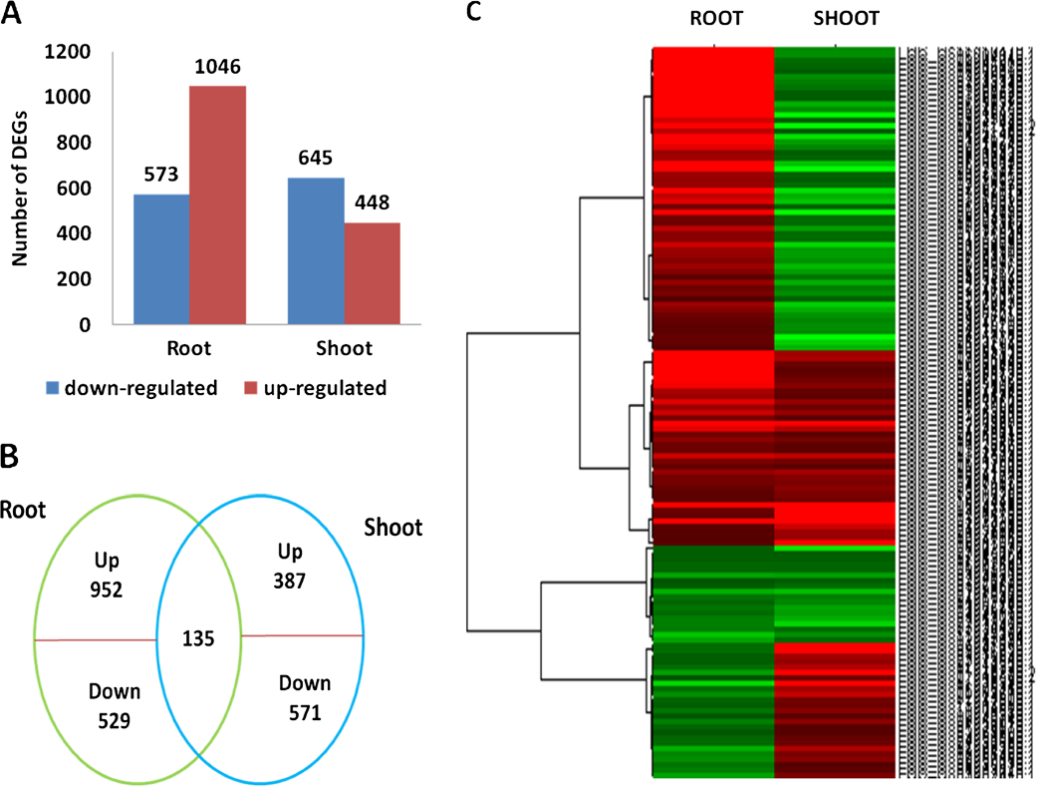
**Differentially-expressed mRNA genes in the root and shoot. (A)** The number of genes up- or down-regulated by Cd treatment by >2-fold in root and shoot (*P* < 0.05); **(B)** Venn diagram showing the unique and shared regulated mRNA genes in rice root and shoot under Cd stress; **(C)** Hierarchical cluster analysis of 135mRNA genes that are regulated in both the root and shoot. The relative fold-changes were analyzed. The fold-change ratios of the genes are indicated by the different colors.

**Figure 4:**
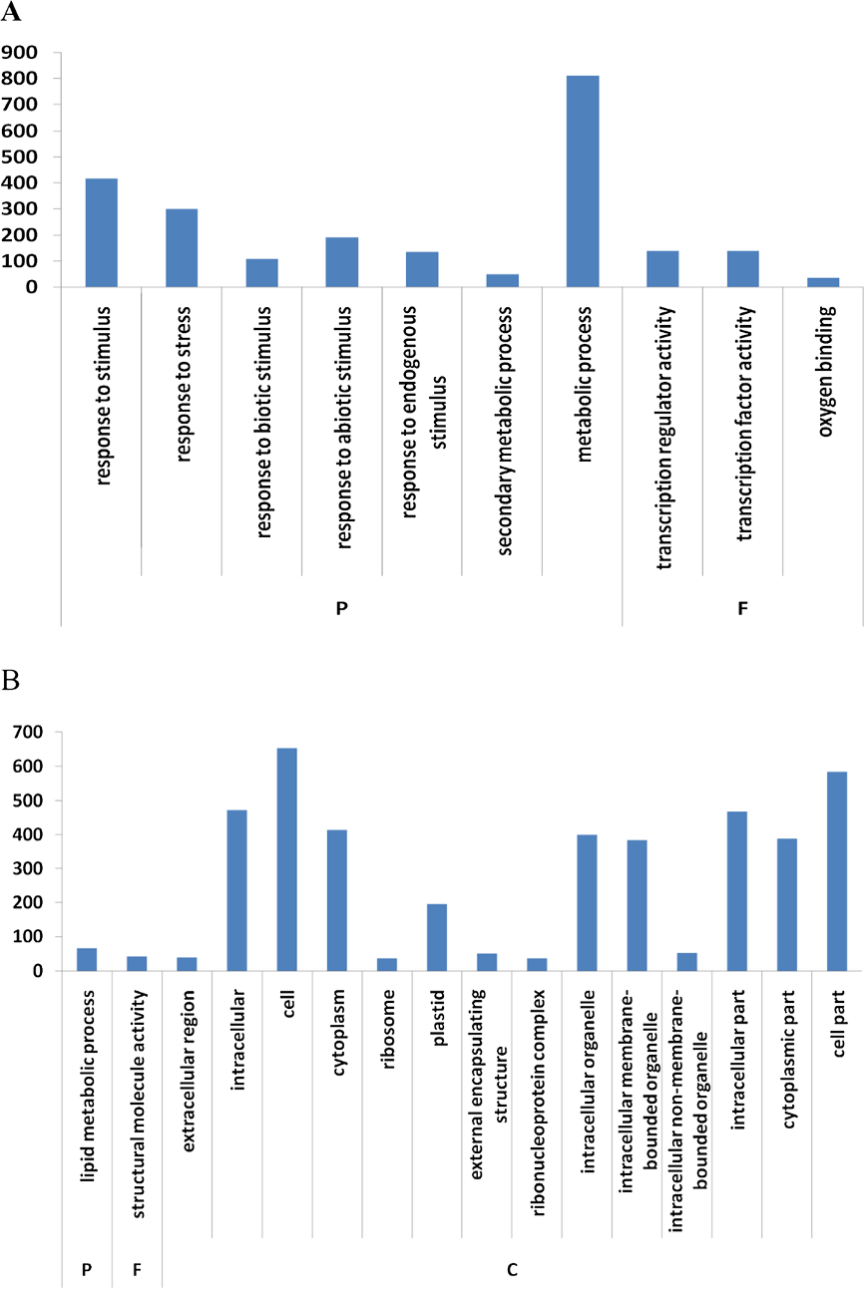
**Gene Ontology (GO) analysis.** biological process **(P)**, molecular function **(F)**, and cellular component **(C)**-of differentially-expressed genes in root **(A)** and shoot **(B)** in response to Cd stress(*P* < 0.05). The y-axis and x-axis indicate the number of genes in a category and the names of the clusters, respectively.

Transcripts detected with at least two-fold differences (|log_2_Ratio| ≥ 1and FDR < 0.001) in the Cd treatment libraries as compared with the control samples were included in our analysis to identify differentially expressed genes. There were 573 down-regulated and 1,046 up-regulated genes in the root, and 645 down-regulated and 448 up-regulated genes in the shoot (Figure 3). These genes from the root and shoot were compared by Venn diagrams to identify genes that showed differences in expression between the two tissues. The number of Cd-responsive genes in both the root and shoot was 135, and these were divided into two groups with four clusters by their expression patterns; 952 genes were specifically up-regulated in the root and 387 specifically in the shoot, while 529 and 571 genes were specifically down-regulated in the root and shoot, respectively.In order to explore the functions of genes that are responsive to Cd treatment in the plant, gene ontology (GO) and pathway enrichment analysis were performed. GO categorization showed that the Cd stress-regulated genes in the root were enriched in metabolic and stress-response processes (Figure 4A), while only genes involved in lipid metabolism were enriched in the shoot (Figure 4B), indicating the differences in the major processes that respond to Cd stress in the two tissues. Two molecular function GO terms, ‘oxygen binding’ and ‘transcription factor activity’, were significantly enriched in the root (Figure 4A), while only ‘structural molecule activity’ was enriched in the shoot (Figure 4B). The genes associated with cytoplasm, ribosomes, plastids, and intracellular organelles were most significant among the cellular component GO terms in the shoot (Figure 4B), while there were no significant cellular component GO terms in the root (Figure 4A).

In an effort to obtain more biological information regarding the molecular and biochemical responses that occur in rice seedlings exposed to Cd treatment, we integrated the Cd-responsive genes set with processes in the KEGG pathway. By applying a cut-off criterion of Q-value <0.05, the enrichment analysis revealed a few important pathways that were significantly enriched in response to Cd stress (Table 3). It was quite evident that, genes involved in certain kinds of secondary metabolite synthesis, such as phenylpropanoids, glutathione, phenylalanine, isoflavonoids, diterpenoids, galactose, carotenoids, amino sugars, and nucleotide sugars, were significantly enriched in the root. We also found that genes for plant hormone signal transduction, protein processing in the endoplasmic reticulum, and ABC transporters were enriched in the root. However, only two pathways, those for ribosome biogenesis and carotenoid biosynthesis, were significantly enriched in the shoot (Table 3). These results indicated that the main responses to Cd stress occurred in the root, and only a few changes, protein and carotenoid biosynthesis, took place in the shoot. The most significantly enriched pathway was plant hormone signal transduction in the root, with 94 genes differentially expressed between the Cd treatment and the control.

**Table 3:** **Significant pathways and proportions after KEGG (Kyoto Encyclopedia of Genes and Genomes) analysis of differentially expressed genes in the root and shoot (Q ≤ 0.05)**

Number	Pathway	DEGs with pathway annotation (866)	All genes with pathway annotation (28280)	P-value	Q-value	Pathway ID
R-1	Plant hormone signal transduction	94 (10.85%)	1560 (5.52%)	2.84E-10	3.15E-08	ko04075
R-2	Phenylpropanoid biosynthesis	48 (5.54%)	596 (2.11%)	1.43E-09	7.93E-08	ko00940
R-3	Glutathione metabolism	23 (2.66%)	193 (0.68%)	3.02E-08	1.12E-06	ko00480
R-4	Phenylalanine metabolism	27 (3.12%)	279 (0.99%)	1.60E-07	4.45E-06	ko00360
R-5	Stilbenoid, diarylheptanoid and gingerol biosynthesis	28 (3.23%)	346 (1.22%)	3.46E-06	7.69E-05	ko00945
R-6	Isoflavonoid biosynthesis	16 (1.85%)	139 (0.49%)	5.89E-06	1.09E-04	ko00943
R-7	Limonene and pinene degradation	21 (2.42%)	228 (0.81%)	8.07E-06	1.28E-04	ko00903
R-8	Protein processing in endoplasmic reticulum	39 (4.5%)	614 (2.17%)	1.70E-05	2.24E-04	ko04141
R-9	Diterpenoid biosynthesis	20 (2.31%)	222 (0.79%)	1.82E-05	2.24E-04	ko00904
R-10	ABC transporters	20 (2.31%)	255 (0.9%)	0.000127	1.42E-03	ko02010
R-11	Flavonoid biosynthesis	23 (2.66%)	351 (1.24%)	0.00057	5.75E-03	ko00941
R-12	Brassinosteroid biosynthesis	9 (1.04%)	81 (0.29%)	0.000826	7.64E-03	ko00905
R-13	Galactose metabolism	13 (1.5%)	155 (0.55%)	0.001002	8.56E-03	ko00052
R-14	Biosynthesis of secondary metabolites	166 (19.17%)	4372 (15.46%)	0.001623	1.29E-02	ko01110
R-15	alpha-Linolenic acid metabolism	13 (1.5%)	170 (0.6%)	0.002303	1.70E-02	ko00592
R-16	Alanine, aspartate and glutamate metabolism	10 (1.15%)	118 (0.42%)	0.003429	2.34E-02	ko00250
R-17	Glycosylphosphatidylinositol(GPI)-anchor biosynthesis	14 (1.62%)	200 (0.71%)	0.003586	2.34E-02	ko00563
R-18	Carotenoid biosynthesis	18 (2.08%)	294 (1.04%)	0.004432	2.73E-02	ko00906
R-19	Amino sugar and nucleotide sugar metabolism	19 (2.19%)	329 (1.16%)	0.006526563	3.81E-02	ko00520
S-1	Ribosome	35 (5.46%)	542 (1.92%)	3.69E-08	3.80E-06	ko03010
S-2	Carotenoid biosynthesis	17 (2.65%)	294 (1.04%)	0.000435344	2.24E-02	ko00906

The hormone-related pathway included auxin, salicylic acid (SA), brassinosteroids (BRs), ethylene (ET), GAs, jasmonate acid (JA) and abscisic acid (ABA) signaling pathway(Additional file 11: Table S7). Most obviously, five PR1 genes and six JAZ genes were up-regulated indicating that the SA and JA signaling pathways were activated. In the BR signaling pathway, six BAK1 and seven BRI1 genes were up-regulated and other five BRI1 genes were down-regulated. Except for three CTR1 genes in ET signaling pathway were down-regulated, another six ERF genes were up-regulated. In the GA signaling pathway, eight GID1 genes were up-regulated and three GID1 genes were down-regulated, four DELLA protein genes up-regulated and one down-regulated. Four SAU1 genes in auxin signaling pathway were up-regulated and two SAU1 genes were down regulated. In addition, we found six PP2C genes and one ABF gene were heavily up-regulated in ABA signaling pathway.

### Combined expression analysis of microRNAs and their target mRNAs during Cd treatment

In our further analysis, we focus on the trend of expression changes of miRNA and its target genes. If a target gene is down-regulated, it suggests that the effective activity of this miRNA is enhanced under the treatment. Vice versa, an up-regulation of a target gene indicates a decrease activity of the corresponding miRNA. Therefore, a miRNA-mRNA interaction pair means anti-regulation of a miRNA and a corresponding mRNA [41, 42]. In order to identify correlations between them, we searched two online databases to find predicted targets in the plant microRNA database (PMRD) (http://bioinformatics.cau.edu.cn/PMRD/) [43] and starBase (http://starbase.sysu.edu.cn/) [44]. There were 9,053 miRNA-mRNA pairs in PMRD, and 2,927 pairs in starBase v2.0, with 1,801 pairs common to the two databases. This large difference in the numbers was due to the fact that most of pairs in starBase were identified based on CLIP-Seq and Degragome-Seq data, while miRNA-mRNA pairs in PMRD were mainly predicted computationally. In order to acquire high quality, complete information regarding miRNA and mRNA expression, we integrated our sequencing data into these two databases.

**Figure 5:**
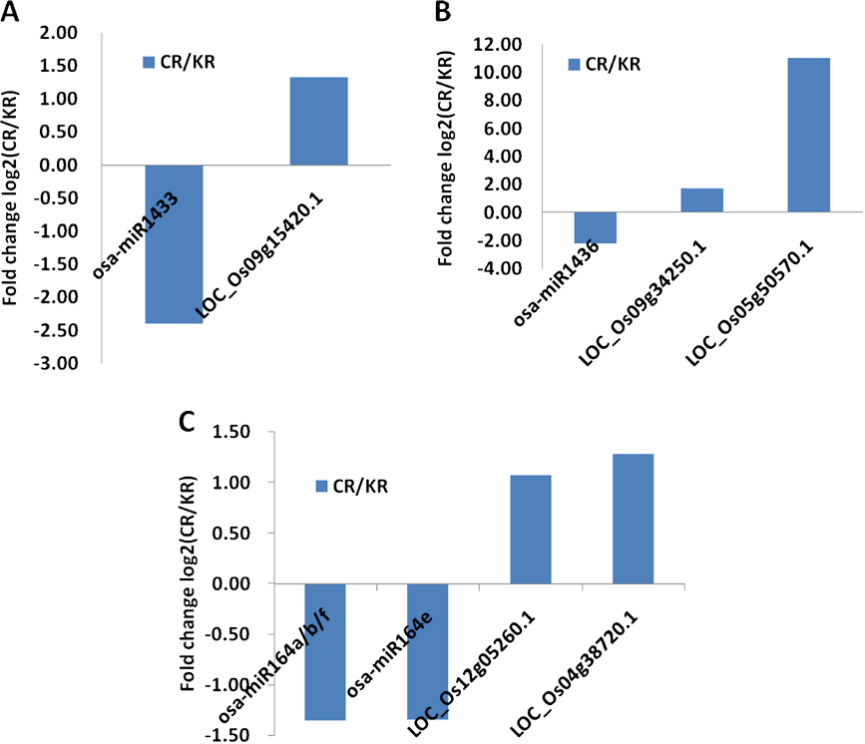
**Representative correlations between miRNAs and mRNAs from two datasets.** Results shown are the fold changes in expression of these transcripts in Cd-treated roots compared to the control. **(A)** The opposite expression pattern of osa-miR1433 and its target LOC_Os09g15420.1; **(B)** The opposite expression pattern of osa-miR1436 and its targets LOC_Os09g34250.1 and LOC_Os05g50570.1; **(C)** The opposite expression pattern of osa-miR164a/b/f, osa-miR164e and their targets LOC_Os12g05260.1 and LOC_Os04g38720.1.

In our miRNA sequencing results, we acquired a total of 141 differentially-expressed miRNAs belonging to 48 different families in the root (Additional file 6: Table S3), and 39 miRNAs belonging to 23 families in the shoot (Additional file 7: Table S4). Based on the two downloaded data sets, we first searched the expression of all different miRNA targets. We then filtered possible targets based on a pre-determined cut-off point (|log2Ratio| ≥ 1 and FDR < 0.001). As a result, we independently acquired 13 miRNA-mRNA pairs from starBase and 30 pairs from PMRD (Additional file 12: Table S8). A Venn diagram showed that 10 of the microRNA-mRNA interaction pairs were acquired from both of the databases (Table 4). One target (LOC_Os09g15420.1) of miR1433 was up-regulated by miRNAs during Cd treatment (Figure 5A), and it is a putative NAD-dependent epimerase/dehydratase family protein. Two targets of miR1436 were also up-regulated in response to Cd treatment (Figure 5B); these were LOC_Os09g34250.1 and LOC_Os05g50570.1, of which LOC_Os09g34250.1 is a predicted UDP-glucoronosyl and UDP-glucosyl transferase domain-containing protein, and LOC_Os05g50570.1 is a putative serine carboxypeptidase homologue. Two targets (LOC_Os04g38720.1 and LOC_Os12g05260.1) of miR164a/b/f and miR164d were also up-regulated in the pairs (Figure 5C). Because miR164a, miR164b, and miR164f share the same sequence, deep sequencing cannot distinguish them, and we thus denoted them as miR164a/b/f (Figure 5C). These two targets are a putative phytosulfokine precursor and NAC (NAM/ATAF1/CUC2) protein, respectively. Although we identified 39 differentially-expressed miRNAs and 1,093 mRNAs in the shoot, no interaction pairs were identified in the above two databases.

**Table 4:** **mRNA targets predicted in common from two databases for differentially expressed miRNAs in root**

miRNA Family	miRNA Name	Target Gene-Interaction	miRNA expression level		mRNA expression level		Targets annotation
			CR/KR-Fold change	P-value	CR/KR-Fold change	P-value	
osa-miR1433	osa-miR1433	09 g15420.1	-2.41	0.00	1.33	0.00	NAD dependent epimerase/dehydratase family protein
osa-miR1436	osa-miR1436	09 g34250.1	-2.15	0.00	1.73	0.00	UDP-glucoronosyl and UDP-glucosyl transferase domain containing protein
	osa-miR1436	05 g50570.1	-2.15	0.00	11.00	0.00	OsSCP29 - Putative Serine Carboxypeptidase homologue
osa-miR164a	osa-miR164a	12 g05260.1	-1.36	0.00	1.07	0.00	phytosulfokines precursor
	osa-miR164a	04 g38720.1	-1.36	0.00	1.29	0.00	no apical meristem protein
	osa-miR164b	12 g05260.1	-1.36	0.00	1.07	0.00	phytosulfokines precursor
	osa-miR164b	04 g38720.1	-1.36	0.00	1.29	0.00	no apical meristem protein
	osa-miR164f	12 g05260.1	-1.36	0.00	1.07	0.00	phytosulfokines precursor
	osa-miR164f	04 g38720.1	-1.36	0.00	1.29	0.00	no apical meristem protein
	osa-miR164e	04 g38720.1	-1.35	0.00	1.29	0.00	no apical meristem protein

**Figure 6:**
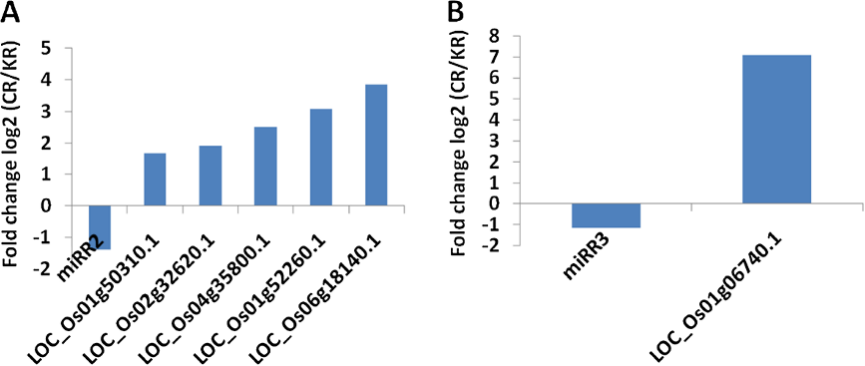
**Representative correlations between new miRNA candidates and predicted mRNA targets.** Results shown are the fold changes in expression of these transcripts in Cd-treated roots compared to the control. **(A)** The opposite expression pattern of miRR2 and its targets LOC_Os01g50310.1, LOC_Os02g32620.1, LOC_Os04g35800.1, LOC_Os01g52260.1 and LOC_Os06g18140.1; **(B)** The opposite expression pattern of miRR3 and its target LOC_Os01g06740.1.

In addition to analyzing the interactions between known miRNAs and mRNAs, we also investigated new miRNA-mRNA interaction pairs in response to Cd stress. Firstly, we predicted new miRNA candidates’ targets (Additional file 13: Table S9) by using a plant target prediction tool available in the University of East Anglia (UEA) sRNA toolkit (http://srna-workbench.cmp.uea.ac.uk/) [34]. After integrating the predicted targets with our sequencing data, we then acquired six miRNA-mRNA interaction pairs showing opposing expression patterns from the root (Table 5 and Additional file 9: Table S6). Five predicted targets (LOC_Os01g50310.1, LOC_Os02g32620.1, LOC_Os04g35800.1, LOC_Os01g52260.1 and LOC_Os06g18140.1) of miRR2 were up-regulated under Cd stress (Figure 6A). These five targets were a putative VIP1 protein, a PAN domain-containing protein, a zinc-finger family protein, a serine acetyltransferase protein, and a UDP-glucoronosyl and UDP-glucosyl transferase domain-containing protein, respectively. Besides, a ribosome-inactivating protein (LOC_Os01g06740.1), a target of miRR3, was also regulated under Cd stress (Figure 6B).

**Table 5:** **Interaction pairs for new miRNA candidates and predicted mRNA targets in root**

sRNA ID	Start-end position of target	Target gene accession	miRNA expression level		mRNA expression level		Targets annotation
			CR/KR-Fold change	P-value	CR/KR-Fold change	P-value	
miRR2	19-39	01 g50310.1	-1.39	0.01	1.67	0.00	VIP1 protein
miRR2	339-357	02 g32620.1	-1.39	0.01	1.90	0.00	PAN domain-containing protein At5g03700 precursor
miRR2	95-114	04 g35800.1	-1.39	0.01	2.49	0.00	zinc finger C-x8-C-x5-C-x3-H type family protein
miRR2	522-540	01 g52260.1	-1.39	0.01	3.06	0.00	serine acetyltransferase protein
miRR2	107-126	06 g18140.1	-1.39	0.01	3.84	0.00	UDP-glucoronosyl and UDP-glucosyl transferase domain containing protein
miRR3	414-432	01 g06740.1	-1.13	0.01	7.05	0.00	ribosome inactivating protein

### Biological repeatability analysis,real-time RT-PCR validation and metabolite changes verification

**Figure 7:**
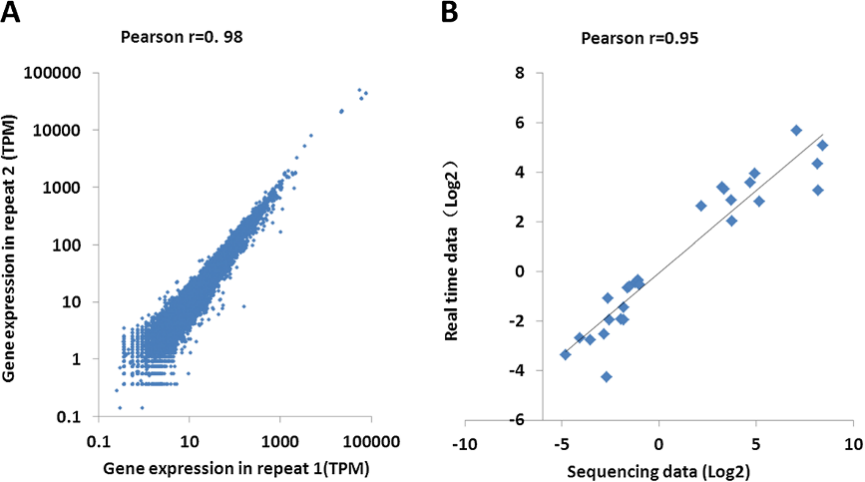
**Correlation of gene expression ratios between the two replicates (A), and between sequencing data and quantitative RT-PCR data (B). (A)** Reproducibility analysis of two independent libraries constructed from root tissue treated with CdCl_2_ for 6 hours. The relativity analysis was based on the TPM from these two libraries. **(B)** Pearson correlation scatters plots of comparisons of ratios measured by sequencing and quantitative RT-PCR in mRNAs and miRNAs. Thirty genes, including 20 mRNAs and 10 miRNAs, were randomly selected and were subjected to quantitative real-time PCR analysis. The rice UBC and U6 RNAs were used as internal standards. Sequencing data (fold changes in gene expression) were plotted against qRT-PCR data (fold-changes in gene expression). Both the x and y-axes are shown in log_2_ scale. r indicates the Pearson correlation coefficient.

To validate our results, we ran biological repeatability analyses based on two independent Cd treatment libraries which were also constructed from root tissue treated with CdCl_2_ for six hours. Scatter plots of TPM (number of transcripts per million clean tags) from the two independent libraries were constructed to explore their relativity, with Pearson correlation values of r = 0.98 (Figure 7A). Eighty-three of 141 differentially-expressed miRNAs were found to have changes in relative expression levels in our second miRNA library; 1,124 of 1,616 differentially-expressed mRNAs were found to be changed in the mRNA library, and six out of 10 miRNA-mRNA interaction pairs were simultaneously identified. These results showed the representation of every library in our analysis.

To validate the expression data further, the relative expression levels of selected genes were investigated with qRT-PCR. We first randomly choose 20 differentially-expressed mRNAs and 10 miRNAs from our sequencing data, and specific primers were used to quantify each gene (Additional file 1: Figure S2; Additional file 2: Table S10). At least three biological replicates and three technical replicates were performed to ensure the quantification of each gene. Correlation between the relative expression level detected by qRT-PCR and by deep-sequencing was calculated. Pearson correlation values were highly significant with r = 0.95 (Figure 7B), which strongly supported the sequencing data. Then, we validated 41 key Cd-responsive genes including transcription factors, kinase, metabolic enzymes and transporters (Additional file 2: Table S10). A similar result was observed with the validation of the key Cd-responsive genes as shown in Additional file 2: Table S10; most of them have the same expression pattern with the sequencing data and confirmed the differences in gene expression patterns during Cd stress.

Based on our transcriptome data, we can conclude that carotenoid biosynthesis was affected in the shoot under Cd stress. To verify the transcriptome result metabolically, we measured the change in ABA content, which is a direct down-stream product of carotenoid metabolism, using an HPLC system. The ABA content in the control was 0.60 ± 0.06 μg/g dry weight. After 6 h of Cd stress, when tissue for our expression data was harvested, the ABA content rose to 0.81 ± 0.10 μg/g dry weights. There was a 34.30% increase in ABA content in comparison with the control (P ≤ 0.01) (Additional file 14: Figure S3). This result independently supports our expression data showing that carotenoid biosynthesis was affected in the early stage of Cd stress in shoots.

## Discussion

The high-throughput sequencing method has become a powerful tool to analyze the expression profiles of genes and identify low-abundance novel miRNAs [24, 45]. Global expression profiling analysis of miRNAs and mRNAs in the same samples may provide a unique opportunity to enhance our understanding of potential miRNA regulatory mechanisms in rice seedlings exposed to Cd. In this study, a total of 146 differentially-expressed miRNAs were identified in the root and 39 in the shoot (Additional file 6: Table S3 and Additional file 7: Table S4). Also, 137, 69, 154, and 165 new miRNA candidates were identified in the KR, CR, KS, and CS libraries, respectively (Additional file 8: Tables S5). The number of differentially-expressed new miRNA candidates was eight in the root and five in the shoot. Previous studies using microarray technology or qRT-PCR to investigate transcriptional regulation of the plant response to Cd stress also identified some differentially-expressed miRNAs. In rice, a total of 19 Cd-responsive miRNAs were identified in Cd-treated rice based on a microarray assay [13]. Ten miRNAs including miR162a, miR168a, miR166e, miR171a, miR171b, miR171g, miR156a, miR156k, miR156l, and miR444b.1 were identified as having the same expression pattern in our study (Additional file 6: Table S3). A previous sequencing study identified 19 novel Cd stress-regulated miRNAs and nine known miRNAs from miRBase in a library of small RNAs from Cd-treated rice seedlings [14]. Six known miRNAs including miR160, miR164, miR167, miR168, miR169, and miR171 were also identified in our study. These limiting but important references show that numerous miRNAs are involved in the Cd stress-response in rice. In *M. truncatula*, miR393, miR171, miR319, and miR529 were up-regulated, whereas miR166 and miR398 were down-regulated in response to Cd treatment as determined by a qRT-PCR-based assay [16]. Compared with these studies, which were limited to known miRNAs, our direct sequencing data detected both known and new miRNA candidates, and thus provided a more comprehensive result.

**Figure 8:**
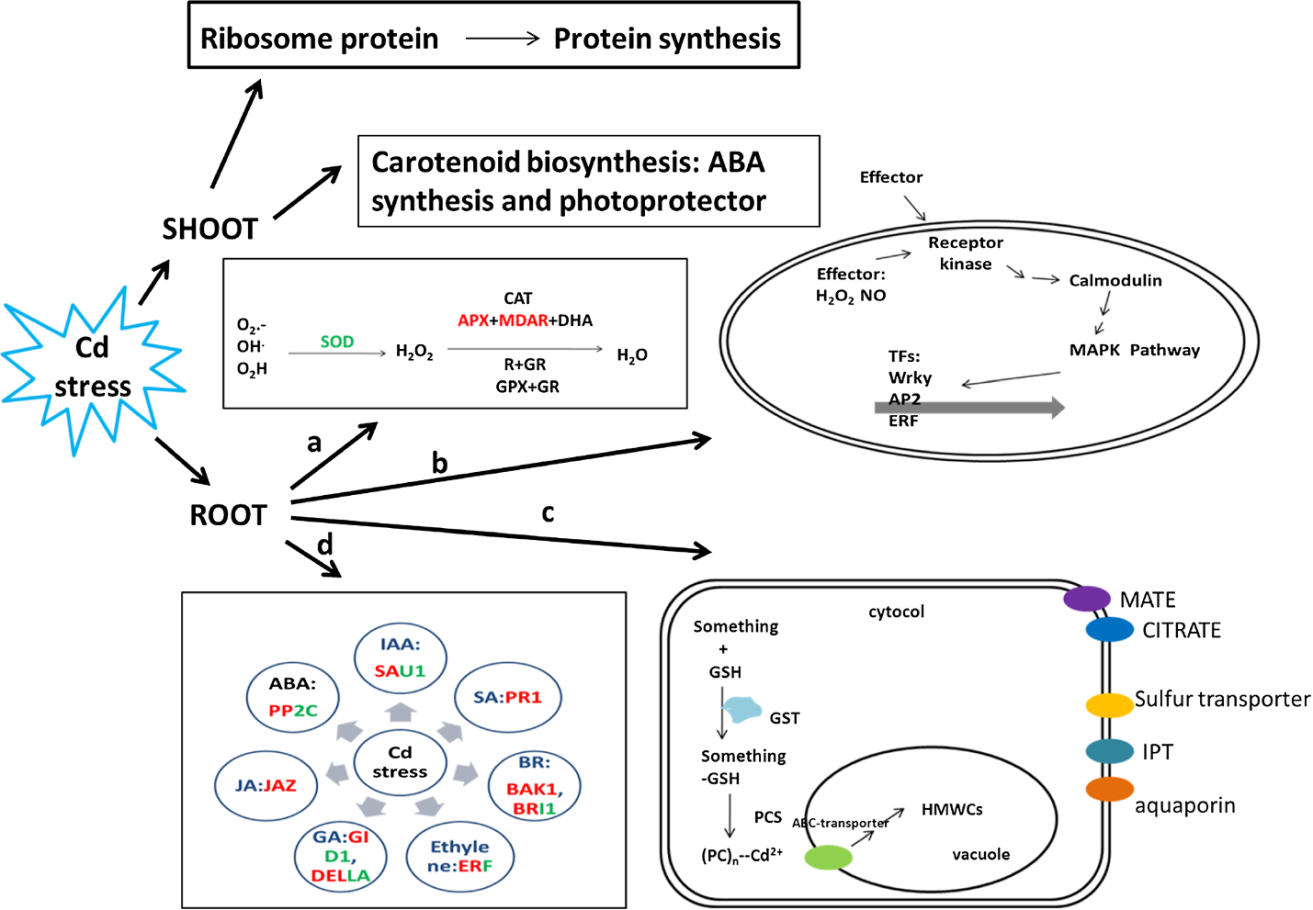
**A simple model was constructed on the basis of transcriptome data.** Under Cd stress, different responses occurred in the root and shoot. In the root, antioxidant system, phosphorylation cascade, plant hormone signaling and detoxify and protection system were activated. In the antioxidant system, key enzymes such as SOD, APX and MDAR were regulated under Cd stress. They were activated to clear hydroxyl radicals (^.^OH), O_2_^-^, ^.^O_2_H and H_2_O_2_. In the phosphorylation cascade, several receptor kinases, calmodulin, MAPK pathway components and some transcription factors, such as Wrky, AP2 and ERF were involved in Cd stress. Several phytohormones such as IAA, SA, BR, Ethylene, GA, JA and ABA signaling pathway were activated or inhibited under Cd stress. In detoxifying and protection system, to transfer Cd complex into vacuole, enzymes involved in GSH production and PC synthesis were heavily expressed. Besides, transporters including MATE, CITRATE, Sulfur transporter, IPT and aquaporin were up-regulated or down-regulated under Cd stress. In the shoot, the most significant change was that the ribosome protein expression was down-regulated, which lead protein synthesis to slow down to cope with Cd stress. Besides, carotenoid biosynthesis was also affected under Cd stress, which would directly affect ABA synthesis and photoproctection.

Under Cd stress, plants respond with several physiological changes:(1) excluding Cd from root absorption by excreting organic acids such as malate or citrate to chelate and immobilize Cd in the soil matrix [46], (2) immobilizing Cd and preventing its uptake into the cytosol by cell wall and extracellular carbohydrates [47], and (3) producing chelating compounds such as phytochelatins or metallothioneins to detoxify and localize Cd to specific cellular compartments [48]. Certainly, plants will produce stress-related proteins and signaling molecules, which will affect plant hormone levels and signal transduction [47]. A simple model was constructed on the basis of our transcriptome data along with the physiological changes under Cd stress (Figure 8). Under Cd stress, different responses occurred in the root and shoot. In the root, the antioxidant system, phosphorylation cascade, plant hormone signaling and detoxification and protection system were activated. In the shoot, the most significant change was the down-regulation of the ribosome protein expression levels and carotenoid biosynthesis.

In our DEG data, we found that synthesis of secondary metabolites such as glutathione, phenylpropanoids, stilbenoids, diarylheptanoids, gingerol, and flavonoids, signaling molecules and ABC transporters were significantly altered by Cd stress (Table 3). This is consistent with previous results that identified genes involved in sulfur assimilation-reduction, glutathione (GSH) metabolism, enzymes catalyzing the biosynthesis of phenylpropanoids, unfolded protein binding, antioxidant responses, and metal transport [17, 23, 49–51]. Based on a fluorescent differential display method, sequences related to signal transduction, protein denaturing stress, and responses to signal transduction were found to be controlled by Cd-responsive genes in *A. thaliana* [51]. Some signaling molecules including transcription factors such as DREB1 (Dehydration-Responsive Element Binding protein) and NAC (NAM, ATAF1/2 and CUC2 domain proteins), and protein kinases were induced in response to Cd stress [23]. In addition, Cd-regulated PDR (plant pleiotropic drug resistance) and MATE (Multidrug and toxic compound extrusion) family transporter genes were strongly up-regulated [23].

The ribosomal proteins are highly conserved components of ribosomal subunits involved in the cellular process of translation [52]. A proteomic study found that ribosome proteins were induced in response to early Cd stress, while repressed under long time stress in *Schizosaccharomyces pombe* [53]. Similarly, we found most ribosomal proteins were down-regulated in our study, indicating that the protein synthesis process was decreased heavily under Cd stress. In fact, the regulation of ribosome biogenesis is required to save energy and nutrients, and to adapt to environment changes [54]. Under Cd stress, reduction in the synthesis of ribosomal proteins is necessary to permit energy and other resources to be distributed to other processes involved in surviving.

In this study, we found that the expression of several key genes in carotenoids synthesis were induced or decreased under Cd stress in both root and shoot. Carotenoids represent the second most abundant pigments in nature. They harvest light for photosynthesis and act as photoprotectors for plant adaptation to high intensity light stress [55]. Previous studies shown that exposure to Cd stress will lead to impairment of the photosynthetic function in many plant species. Both chlorophylls and carotenoid contents decrease under Cd exposure [48, 56, 57]. In our study, ABA content was increased and its signaling pathway was activated. Oxidative cleavage of carotenoids will produce apocarotenoids, while the phytohormone ABA is an apocarotenoid derivative [58]. ABA was found taking part in the regulation of antioxidative defense systems and Cd-induced oxidative stress in mung bean seedlings [59]. All these results suggest that both the synthesis of carotenoids and the ABA signaling pathway were conservatively involved in the plant Cd stress response.

Previous studies have shown that plants can activate the expression of antioxidant enzymes against the occurrence of activated oxygen and oxidative injury caused by Cd stress [17–19], and these biological processes were also observed in our analysis (Table 3; Figure 8). Importantly, these activities may be directly associated with tolerance to stress. For example, rice Xiushui 110, which is highly tolerant to Cd toxicity, had the greatest increase in SOD and POD activities. In contrast, rice variety Bing 9914, which is sensitivity to Cd toxicity, had the least increase in activities of antioxidative enzymes [60]. Therefore, investigating the Cd response activity has great significance to understand the plant tolerance mechanism and may benefit the selection of plant varieties for phytoremediation.

In general, increased miRNA activity will lead to the down-regulation of an mRNA target, while decreased miRNA activity will lead to up-regulation of the target. Though miRNAs regulate target gene expression by repressing their targets through transcript cleavage or translation repression [4, 61, 62], integrated analysis of miRNA and mRNA expression profiles can still be helpful to identify the functional miRNA-mRNA interaction pairs involved in regulating specific biological processes [63]. After integrated analysis of differentially expressed miRNAs and mRNAs, we found several important regulatory miRNAs involved in Cd stress. MiR1433 appeared to target NAD-dependent epimerase/dehydratase family proteins in response to Cd treatment (Figure 5A). Previous studies showed that this family of proteins utilizes NAD as a cofactor to take part in a variety of chemical reactions [64], suggesting that various metabolic changes occur during Cd stress, and the down-regulation of miRNAs leads to the rapid production of this kind of protein to catalyze different metabolic processes. MiR1436 targets UDP-glucoronosyl and UDP-glucosyl transferase domain-containing proteins and a putative serine carboxypeptidase homologue (Figure 5B). Combined with the NAD-dependentepimerase/dehydratase family protein, we can conclude that the most significant changes in response to Cd stress were metabolic processes. Other than metabolic changes, a NAC family transcription factor, a target of miR164, was also up-regulated due to Cd treatment (Figure 5C). One new miRNAs, miRR2, appeared to target one zinc-finger family protein (Table 5 and Figure 6). Previous results have shown that numerous transcription factors are involved in the Cd response [65, 66]. Five miRNA families (miR166, miR171, miR396, miR156, and miR444), whose target genes encode transcription factors, were all down-regulated in response to Cd exposure in rice [13].

In this study, we detected 146 miRNAs that showed differential expression in response to Cd stress in the root and 39 in the shoot. However, only six miRNAs were identified that form a total of 10 miRNA-mRNA interaction pairs. This number is far below our expectation that more interaction pairs would be found from the 146 miRNAs. Possible reasons to explain this phenomenon include: (1) the commonly-predicted targets in the two online databases do not represent the actual existing interactions, (2) plants can regulate the expression of specific genes at the temporal and spatial levels, and most targets may not be expressed at this point, and (3) the accepted standard used for the definition of differentially-expressed genes may miss some interactions, and more interaction pairs may be identified by lowering the threshold. We found that there were no interaction pairs in the shoot, and the responsive miRNAs and mRNAs were fewer than in root, especially the number of miRNAs. A time-course analysis of gene regulation under Cd stress in rice found that, compared with the root, the shoot had fewer Cd-responsive genes when exposed to 1 μM Cd for 24-72 h [23], which supports our results. When the exposure time was extended to eight days, the number of Cd-regulated genes in shoots was larger than it was in roots. Therefore, the treatment time and concentration may explain the differential gene expression patterns seen in the root and the shoot.

## Conclusions

Four libraries were constructed, amplified and sequenced with root and shoot tissues from 7-day-old rice seedlings exposed to solutions with and without 60 μM CdCl_2_ for 6 h. 141 known miRNAs belonging to 48 families and 39 known miRNAs in 23 families were identified to be differentially expressed in the root and the shoot, respectively. In addition, we identified eight new miRNA candidates from the root and five from the shoot that were differentially expressed in response to Cd treatment. For the mRNAs, we identified 1,044 genes in the root and 448 genes in the shoot that were up-regulated, while 572 and 645 genes were down-regulated in the root and shoot, respectively. GO and KEGG enrichment analyses showed that genes encoding secondary metabolite synthases, signaling molecules, and ABC transporters were significantly enriched in the root, while ribosomal protein and carotenoid biosynthesis genes were significantly enriched in the shoot. We identified 10 known and six new miRNA-mRNA interaction candidate pairs that showed significant inverse expression patterns. This work provides an important advance in the functional identification of miRNAs and how they interact with their targets in response to Cd treatment. Studies on each interaction pair will provide more fundamental information about how plants respond to Cd stress at the molecular level.

### Availability of supporting data

The raw reads reported in this study have been deposited in the National Center for Biotechnology Information Short Reads Archive (http://www.ncbi.nlm.nih.gov/sra website) under accession number SRP045693.

## Electronic supplementary material

Additional file 1: Figure S2: Validation of sequencing data by qRT-PCR. (DOCX 22 KB)

Additional file 2: Table S10: Validation data for several key Cd-responsive genes. (XLSX 20 KB)

Additional file 3: Table S11: Oligonucleotide primers used in Real-time PCR assays in this study. (XLSX 11 KB)

Additional file 4: Table S1: Sequencing results for small RNAs from four libraries. (XLSX 11 KB)

Additional file 5: Table S2: Distribution of small RNAs among 11 different categories. (XLSX 11 KB)

Additional file 6: Table S3: Differentially-expressed miRNAs in response to Cd stress in root libraries. (XLSX 17 KB)

Additional file 7: Table S4: Differentially-expressed miRNAs in response to Cd stress in shoot libraries. (XLSX 12 KB)

Additional file 8: Table S5: Identified new miRNA candidates in library KR, CR, KS and CS. (XLSX 110 KB)

Additional file 9: Table S6: The basical information for the precursor of the new miRNAs' candidates. (XLSX 12 KB)

Additional file 10: Figure S1: The accumulation of genes mapped by all clean tags and unique clean tags in five libraries. (DOCX 186 KB)

Additional file 11: Table S7: List of DEGs in phytohormone signaling pathways. (XLSX 17 KB)

Additional file 12: Table S8: Predicted targets for differentially expressed miRNAs based on starBase and miRBase database searches in root and shoot. (XLSX 12 KB)

Additional file 13: Table S9: The predicted targets for the new miRNA candidates using UEA sRNA toolkit. (XLSX 21 KB)

Additional file 14: Figure S3: The change of ABA content under Cd stress in shoots. (DOCX 18 KB)
