# Supplementary material for: Integrated analysis of miRNA and mRNA expression profiles in response to Cd exposure in rice seedlings
Source: BMC Genomics. 2014 Oct 1;15(1):835. doi: 10.1186/1471-2164-15-835 (PMC4193161; doi:10.1186/1471-2164-15-835)
Supplement: Supplementary file 1 — Additional file 1: Figure S2: Validation of sequencing data by qRT-PCR. (DOCX 22 KB) [file 12864_2014_6517_MOESM1_ESM.docx]

Figure S2 Validation of sequencing data by qRT-PCR. Thirty genes, including 20 mRNAs and 10 miRNAs, were randomly selected and were subjected to quantitative real-time PCR analysis. The rice UBC and U6 RNAs were used as internal standards, respectively. The fold change of expression is the gene expression level in Cd treatment normalized to that in CK. Bars depict SD (n=3).
