# Supplementary material for: Integrated analysis of miRNA and mRNA expression profiles in response to Cd exposure in rice seedlings
Source: BMC Genomics. 2014 Oct 1;15(1):835. doi: 10.1186/1471-2164-15-835 (PMC4193161; doi:10.1186/1471-2164-15-835)
Supplement: Supplementary file 10 — Additional file 10: Figure S1: The accumulation of genes mapped by all clean tags and unique clean tags in five libraries. (DOCX 186 KB) [file 12864_2014_6517_MOESM10_ESM.docx]

**Supporting Information**


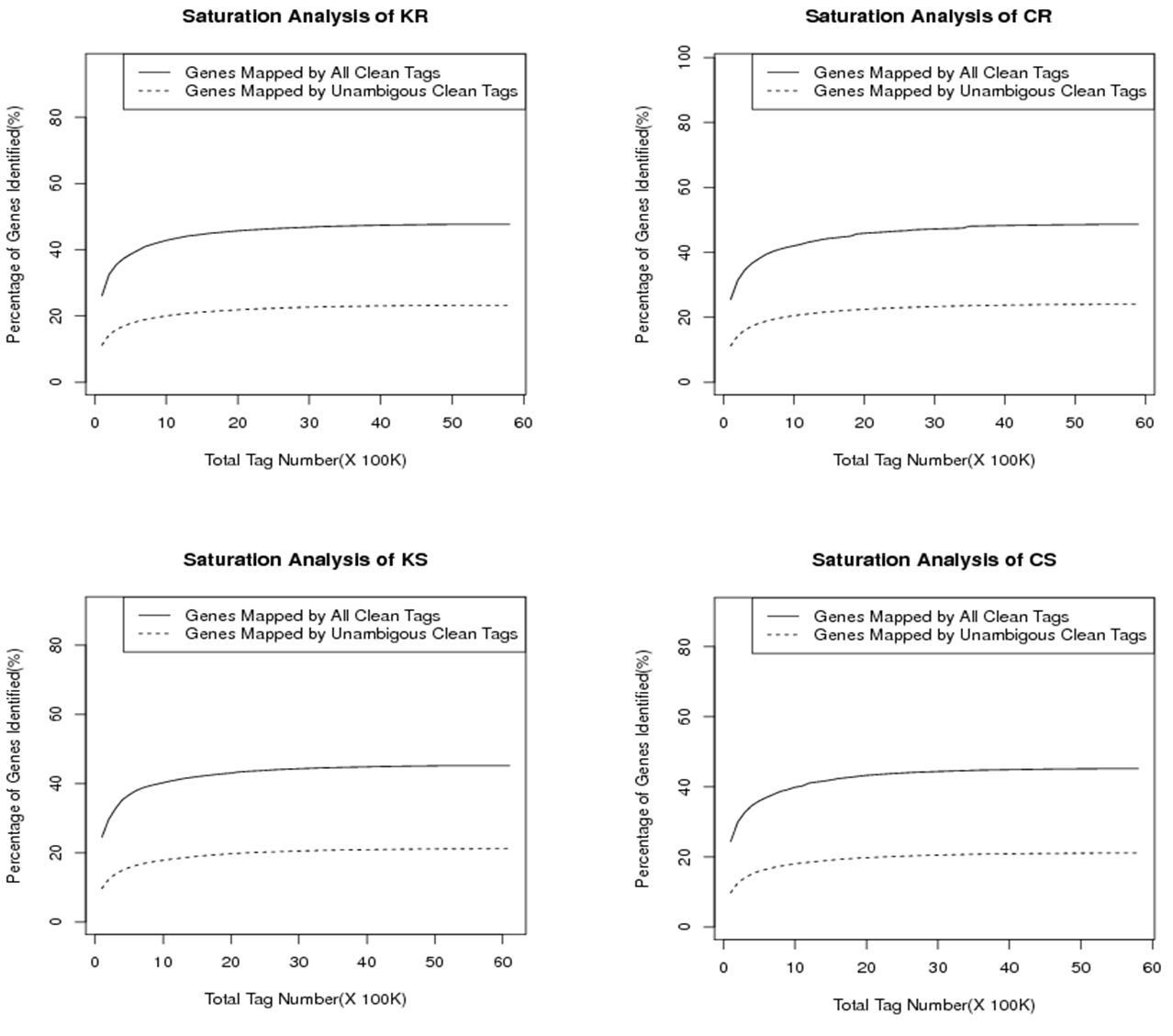


Figure S1.The accumulation of genes mapped by all clean tags (solid line) and unique clean tags (broken line) in five libraries. The percentage of genes identified (y-axis) increases as the total number of tags (x-axis) increases.
