# Supplementary material for: Integrated analysis of miRNA and mRNA expression profiles in response to Cd exposure in rice seedlings
Source: BMC Genomics. 2014 Oct 1;15(1):835. doi: 10.1186/1471-2164-15-835 (PMC4193161; doi:10.1186/1471-2164-15-835)
Supplement: Supplementary file 14 — Additional file 14: Figure S3: The change of ABA content under Cd stress in shoots. (DOCX 18 KB) [file 12864_2014_6517_MOESM14_ESM.docx]

Figure S3. The change of ABA content under Cd stress in shoots. Seven-day-old seedlings were treated with and without 60uM CdCl_2_ for 6h. The shoot tissues were crushed in liquid nitrogen and extracted with 8 ml of methyl alcohol-water-acetic acid (15:4:1, v/v), and re-extracted with 5 ml extraction. After separation and purification, the samples were dried totally and dissolved in methyl alcohol-water (80:20, v/v). ABA content was detected by an AGilent1260 HPLC system.
